# GitHub enables collaborative and reproducible laboratory research

**DOI:** 10.1371/journal.pbio.3003029

**Published:** 2025-02-14

**Authors:** Katharine Y. Chen, Maria Toro-Moreno, Arvind Rasi Subramaniam

**Affiliations:** Basic Sciences Division and Computational Biology Section of the Public Health Sciences Division, Fred Hutchinson Cancer Center, Seattle, Washington, United States of America

## Abstract

GitHub, a platform widely used in software development, offers a robust framework for documenting all activities of laboratory research projects. This Community Page highlights the benefits of, and provides guidance for, incorporating the GitHub ecosystem into ‘wet’ lab workflows.

## Introduction

Laboratory research is a complex, collaborative process that involves several stages, including hypothesis formulation, experimental design, data generation and analysis, and manuscript writing. Although reproducibility and data sharing are increasingly prioritized at the publication stage, integrating these principles at earlier stages of laboratory research has been hampered by the lack of broadly applicable solutions. Lab notebooks are the most common media used to document research, but they are typically only used for recording methods and data. Electronic lab notebooks, despite their popularity, are stored in proprietary formats, incur a recurrent cost, tend to become defunct over time, and have poor interoperability with each other [1]. Cloud-based tools like Google Docs and Dropbox allow sharing of data and documents, but do not provide a structured way to track changes over time or record project-related communication. Email and messaging tools such as Slack and Microsoft Teams facilitate informal discussion of ideas and data, but these are poorly suited for organizing data and discussion in a reproducible manner. Consequently, research information often becomes fragmented across multiple platforms. Here, we introduce GitHub as a software platform that can overcome these limitations, and be used across all stages of laboratory research.

## GitHub for laboratory research

**Fig 1:**
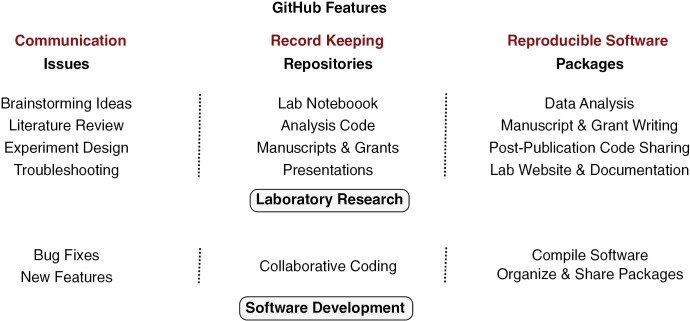
Features of GitHub that are useful for laboratory research and how they compare to software development.

The process of software development bears several similarities to activities in laboratory research; it involves iterative problem-solving, where hypotheses are broken into smaller, testable components, implemented through code, analyzed, and refined as needed. The need to document and share all stages of software development has led to tools and workflows that ensure reproducibility and enable seamless collaboration. Many of these tools and common workflows associated with software development are implemented in GitHub, an online platform where people can store, organize, and share their projects. In the scientific community, GitHub is used to share data analysis workflows after publication [2,3], develop and share computational tools [4], perform individual record keeping [5,6], and conduct open science and collaborative projects [7–11]. However, how the standard workflows and features of GitHub can be adapted to improve reproducibility and collaboration within a traditional laboratory research group has not been explored. We outline below a three-part approach for incorporating the GitHub ecosystem into laboratory research workflows (Fig 1). For a more detailed guide on implementing this approach in a molecular biology laboratory, see the full preprint [12]. In addition, an example GitHub repository based on this approach is available at https://github.com/rasilab/github_demo and a template repository that can be copied is available at https://github.com/rasilab/github_template.

### Designing and organizing experiments using “issues”

Software developers use “issues” on GitHub to track tasks, problems, or ideas related to their project. Each issue serves as a to-do item where team members can describe the problem, propose solutions, and discuss progress in one place. In our research group, we use this feature as an interface to organize and collaborate on all aspects of a laboratory experiment (see preprint [12] for example screenshots). Each experiment begins with the creation of a new issue in the corresponding project repository by any of the project members. The issue is initially used to outline the rationale and background of the experiment and the strategy for performing the experiment. Project members can discuss aspects of experimental design, provide clarification in the comments section, and update the issue description as needed. During the experiment, we use the comments section to discuss troubleshooting steps, intermediate data and figures, and interpretation of results. Once the experiment concludes, we update the issue with key results, figures, and conclusions, turning it into a concise summary of the experiment. Thus, each issue functions as a “gist” of the experiment, easily accessible to all collaborators. The issue number provides a convenient way to reference the experiment across physical samples, work logs, computer file names, and discussions in other issues. GitHub provides a number of useful features such as labels, assignees, milestones, and project boards to organize and prioritize issues within a project and across projects.

### Documenting experiments and data analyses with version control

**Fig 2:**
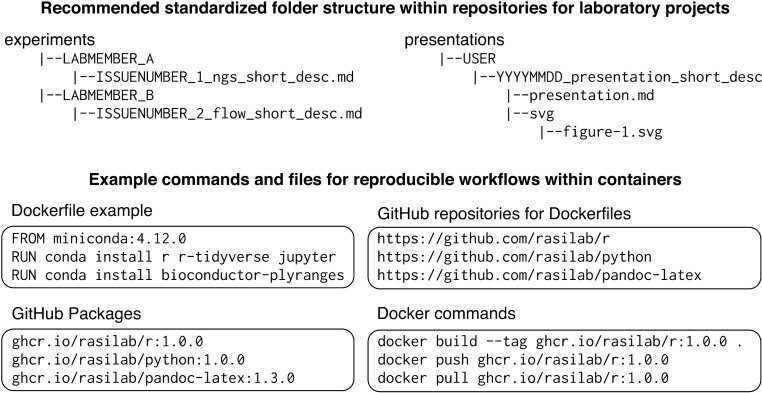
Examples of folder structures and container-related commands and files for reproducible research. A GitHub repository is a cloud-based folder where you can store your code, files, and each file’s version history. A Dockerfile is a simple set of instructions that describe how to set up a software environment on a specific operating system. Docker commands are used to manage Docker containers and images on the local computer.

Git is a version control system that records the history of file additions and modifications in a folder, and is used by programmers to track changes to their code. In our research group, we store all files relevant to a project within a single folder on our local computers. We use Git to track changes in that folder, and synchronize it with a cloud-based GitHub repository. We write documents in plain text to enable interoperability across different software and platforms, and to facilitate version control with Git. Within each repository, we use standardized subfolder names for lab notebook entries, code, data, manuscripts, grants, and presentations (Fig 2). We record all work pertinent to an issue in lab notebook files, similar to traditional lab notebook entries. Each lab notebook file includes the corresponding issue number in its name and a link to the issue in its contents to enable easy cross-referencing.

### Ensuring reproducible software environments with containerized packages

Replicating data analysis workflows and software environments is a common challenge in both software development and laboratory research. Software containers are portable environments that package all the necessary software, libraries, and dependencies for an analysis, ensuring it runs consistently across different computers [13]. In our research group, we use software containers to perform all data analyses and writing tasks in reproducible software environments. Public container registries, such as Docker Hub and BioContainers [14], offer ready-made containers that can be used without installing software, simplifying data analysis. For custom containers, we take advantage of the Packages feature of GitHub to host our containers in a centralized location that is free to use and publicly accessible. Each container in our group’s GitHub Packages collection is linked to a dedicated GitHub repository to store the recipe for creating that container. Our group uses containers in several ways for interactive data analyses, writing tasks, and complex bioinformatic workflows. Containers in our group’s GitHub Packages can also be used by external collaborators and readers of our published manuscripts to reproduce data analyses.

## Benefits of GitHub for “wet” lab research

We recognize that adopting the approach outlined here may involve a steep learning curve, particularly for laboratory research groups with limited computational experience. However, we have provided example repositories, tutorials, and templates to assist with this, and we believe the following benefits make the transition more manageable and outweigh the initial effort—particularly for young labs that are still establishing their workflows: (1) Git and GitHub have comprehensive and user-friendly documentation (see resources above). (2) The workflow and features described here are highly modular and can therefore be incrementally adopted. For example, wet lab teams can start with using GitHub Issues to discuss ideas and experimental design in a structured manner. (3) Researchers can then learn to use Git and GitHub to record their work and results. Postdocs transitioning into faculty jobs can start by building their lab website using GitHub. (4) The features described here are part of the free GitHub tier, and can be used by any research group regardless of their size, funding level, or institutional affiliation. (5) Git and GitHub are widely used in both academia and industry, and thus the organization and documentation practices we describe are highly transferrable skills for trainees.

## Limitations of GitHub

Our approach does not directly address data storage since GitHub is not suitable for storing large data sets. While we provide some solutions in our preprint [12] (see “Use Git to store and track your work”), data storage solutions are ultimately lab- and data type-specific and beyond the scope of this article. Further, GitHub is not suitable for storing sensitive data, as it might breach institutional guidelines. Platforms similar to GitHub such as GitLab and Bitbucket might be more suitable for certain labs to meet their privacy or hosting requirements. GitHub private repositories allow fine-grained access control, but researchers should be aware that information stored on GitHub might be used for training large machine-learning models. Despite these limitations, we find that GitHub can serve as an effective platform for improving reproducibility and collaboration in many wet lab research scenarios.

## Conclusions

Here, we have introduced GitHub and highlighted how this platform can be effectively used to support laboratory research. We have adopted widely used features from software development workflows, such as issues, version control, and containers, and adapted them to the specific needs of a molecular biology laboratory. The versatility, scalability, and affordability of this approach make it suitable for various scenarios, ranging from small research groups to large, cross-institutional collaborations. Adopting this framework from a project’s outset can increase the efficiency and fidelity of knowledge transfer within and across research laboratories.
